# First‐Principles Design of Room Temperature Ferromagnetic Metallic Rare‐Earth Zintl Compounds AB_2_C_2_ (A = Ce, Pr, Nd; B = Li; C = Sb) for Next‐Generation Spintronic and Magneto‐Electronic Applications

**DOI:** 10.1002/open.70225

**Published:** 2026-06-17

**Authors:** Hayat Ullah, Maria Jilani, Ashfaq Ahmad, Asif Mahmood, Sadia Yasin, Uzma Hameed, Akif Safeen, Basit Ali

**Affiliations:** ^1^ Department of Physics Material Modeling and Simulation Lab Women University of Azad Jammu & Kashmir Bagh Pakistan; ^2^ Department of Chemistry Women University of Azad Jammu & Kashmir Bagh Pakistan; ^3^ Chemical Engineering Department College of Engineering King Saud University Riyadh Saudi Arabia; ^4^ Department of Physics, University of Karachi, Karachi Pakistan; ^5^ Department of Physics University of Poonch Rawlakot Rawlakot Pakistan; ^6^ Department of Chemistry and Materials Science School of Chemical Engineering Aalto University Aalto Finland

**Keywords:** Curie temperature, electronic properties, exchange interactions, ferromagnetism, magneto‐electronics, metallicity, room‐temperature ferromagnets, spintronics, structure stability, Zintl phases

## Abstract

A comprehensive density functional theory (DFT) study investigates the structural, electronic, and magnetic properties of rare‐earth Zintl compounds AB_2_C_2_ (A = Ce, Pr, Nd; B = Li; C = Sb). Calculations employing the FPLAPW method (WIEN2k code) utilize PBE‐GGA and GGA + U approximations to accurately treat correlated 4f electrons. Structural optimization reveals ferromagnetic (FM) ordering as the most stable ground state for all compounds, with optimized lattice parameters aligning with lanthanide series trends. Electronic structure analysis confirms metallic behavior driven by rare‐earth 4f and 5d states near the Fermi level. Density of states analysis shows significant hybridization between Sb‐p and RE‐d states, indicating covalent interactions within the anionic [Sb] networks consistent with the Zintl concept. Magnetic characterization reveals total moments localized on rare‐earth sites, with strong FM exchange interactions stabilizing the ground state. Calculated Curie temperatures (T_C_) from DFT + U (with U_eff_  = 0.52–0.58 Ry) and empirical relations exceed room temperature, despite DFT being a 0K method, the predicted T_C_  > 300 K indicates that ferromagnetic order persists under ambient conditions comparable to values reported for spintronic Heusler alloys. Negative formation and cohesive energies confirm thermodynamic stability and experimental viability. In conclusion, these compounds exhibit a promising combination of robust room‐temperature ferromagnetism, metallic conductivity, and structural stability, fundamental prerequisites for spintronic materials requiring efficient spin‐polarized transport, identifying these Zintl phases as compelling candidates for experimental exploration.

## Graphical abstract

DFT calculations (FPLAPW+GGA+U) on tetragonal AB_2_C_2_ (A=ce, pr, Nd; B=Li; C=sb) zintl compounds reval ferromagnetic metallic ground states with curie temperature exceeding 300K (up to 1389K for NdLi_2_Sb_2)_ identifying these materials as promising candidates for spin‐polarized current sources in next generation spintronic devices.

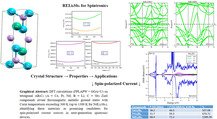

## 1 Introduction

The term “Zintl phase” honors the pioneering work of German chemist Eduard Zintl in solid‐state chemistry [1, 2, 3, 4, 5]. These compounds, occupying a unique electronic position within intermetallic systems, are characterized by a distinctive blend of ionic and covalent bonding [6, 7, 8]. Typically formed by combining electropositive alkali/alkaline earth metals with more electronegative p‐block elements, Zintl phases involve a formal electron transfer from cations to anions, resulting in anionic frameworks that obey valence rules like the (8‐N) rule [9, 10, 11, 12]. The “Zintl border” between Groups 13 and 14 demarcates elements whose combinations often yield these fascinating phases [13, 14]. In recent decades, Zintl phases have garnered significant attention due to their rich chemistry and promising technological applications, leading to a vast array of new compounds [15, 16]. These exhibit a remarkable spectrum of physical properties, including thermoelectricity [17, 18], superconductivity [19], topological insulator behavior [20, 21], and various magnetic phenomena [22, 23]. Antimony is useful in catalysis, degradation, and organic synthesis [24, 25], whereas this work focuses on its electronic and magnetic properties in solid‐state Zintl phases. Notably, magnetic Zintl phases containing transition metals or rare‐earth elements have become a focus for spintronics research. Recent computational studies have revealed intriguing magnetic properties in various Zintl families, including half‐metallic ferromagnetism in XCr_2_Bi_2_ (X = Ca, Sr) [26], complex magnetic ordering in CaCr_2_Sb_2_ [27], and promising thermoelectric and magnetic behavior in KCr_2_L_2_ (K = Ca, Sr; L = P, As) [28]. Other AB_2_C_2_‐type Zintl phases have also been investigated, such as XCu_2_P_2_ (X = Ca, Sr) which exhibit interesting magneto‐electronic properties [29], and XMn_2_Y_2_ (X = Ca, Sr; Y = P, As) showing antiferromagnetic behavior [30].

Particularly relevant to the present work are studies on rare‐earth containing Zintl phases. Computational investigations of compounds like EuIn_2_As_2_ have revealed potential intrinsic magnetic topological insulator behavior [31], while studies on GdSbTe have shown topological nodal line semimetal characteristics [19]. Other rare‐earth Zintl phases like EuGa_2_P_2_ have demonstrated interesting magnetic properties [32], and the Ca14AlSb11 structure type has shown remarkable crystal chemistry with magnetic and thermoelectric properties [23]. The structural, electronic, optical, and elastic properties of Zintl‐phases AE_3_GaAs_3_ (AE = Sr, Ba) have also been systematically studied [33]. These studies collectively demonstrate the rich potential of rare‐earth Zintl phases for multifunctional applications. Concurrently, rare‐earth (RE) elements are critical components in modern technology [34, 35]. Their value stems from unique properties derived from partially filled 4f orbitals, making them indispensable in applications ranging from permanent magnets to spintronics [36, 37]. The partially filled 4f shells give rise to large magnetic moments and significant spin–orbit coupling, properties that are essential for advanced spintronic devices. Beyond rare‐earth systems, layered van der Waals ferromagnets such as CrGeTe_3_ have also emerged as promising spintronic materials [38], further expanding the landscape of magnetic 2D materials. Integrating rare‐earth cations into Zintl phases presents a powerful strategy for designing novel materials, as the localized 4f moments can introduce and tune magnetic functionality within the covalently bonded anionic networks [39].

Despite growing interest in both Zintl chemistry and rare‐earth‐based functional materials, a systematic understanding of rare‐earth‐containing Zintl phases remains incomplete. Specifically, the ternary compounds AB_2_C_2_ (A = Ce, Pr, Nd; B = Li; C = Sb) represent an intriguing yet underexplored family within the broader landscape of these Zintl phases. While isostructural compounds like CaAl_2_Si_2_ [1] and BaCu_2_Sb_2_ [36] have been studied, and while basic synthesis of AB_2_C_2_ (A = Ce, Pr, Nd; B = Li; C = Sb) compounds has been reported, a comprehensive first‐principles investigation of their fundamental physical properties, structural stability, electronic structure, magnetic ground state, and the governing exchange interactions is notably absent. This gap is particularly significant given that recent studies on similar compounds like XCr_2_Bi_2_ [26] and CaCr_2_Sb_2_ [27] have revealed promising spintronic properties. The role of the correlated 4f electrons in governing these properties, particularly in dictating magnetic order and potential metallic behavior, remains unknown. The discovery of room‐temperature ferromagnetic metals with high spin polarization is crucial for advancing spintronic technologies including spin valves, magnetic tunnel junctions, and spin‐transfer torque devices [40, 41, 42]. Machine learning accelerates materials discovery in adjacent fields [43 **,** 44] and could complement DFT studies of Zintl phases in the future.

This knowledge gap motivates our computational study. Using density functional theory (DFT) with the full‐potential linearized augmented plane wave (FPLAPW) method, we perform the first detailed analysis of AB_2_C_2_ (A = Ce, Pr, Nd; B = Li; C = Sb). We employ both standard generalized gradient approximation (GGA) and the GGA + U approach to accurately model the electron correlation in the 4f shells, following methodologies successfully applied to similar correlated electron systems [45, 46, 47, 48]. Our objectives are to (i) determine their ground‐state structural and magnetic stability, (ii) analyze their electronic structure and bonding nature, (iii) characterize their magnetic properties including atomic moments and the strength of exchange interactions, (iv) predict key stability (formation energy) and magnetic (Curie temperature) parameters, and (v) assess their potential for spintronic and magneto‐electronic applications by comparing with similar compounds reported in literature. The primary applied motivation lies in spintronics, which requires materials capable of efficient spin generation, manipulation, and detection [35, 37]. Intrinsic ferromagnetic metals are particularly desirable as sources of spin‐polarized currents. By elucidating whether these AB_2_C_2_ (A = Ce, Pr, Nd; B = Li; C = Sb) compounds are thermodynamically stable, metallic, and ferromagnetic with strong exchange coupling, this work aims to identify their potential as a new class of materials for spintronic device architectures. Our findings are intended to provide essential theoretical guidance for future experimental synthesis and property characterization, thereby contributing to the targeted design of functional rare‐earth Zintl phases and expanding the family of promising spintronic materials.

## 2 Computational Methodology

**FIGURE 1:**
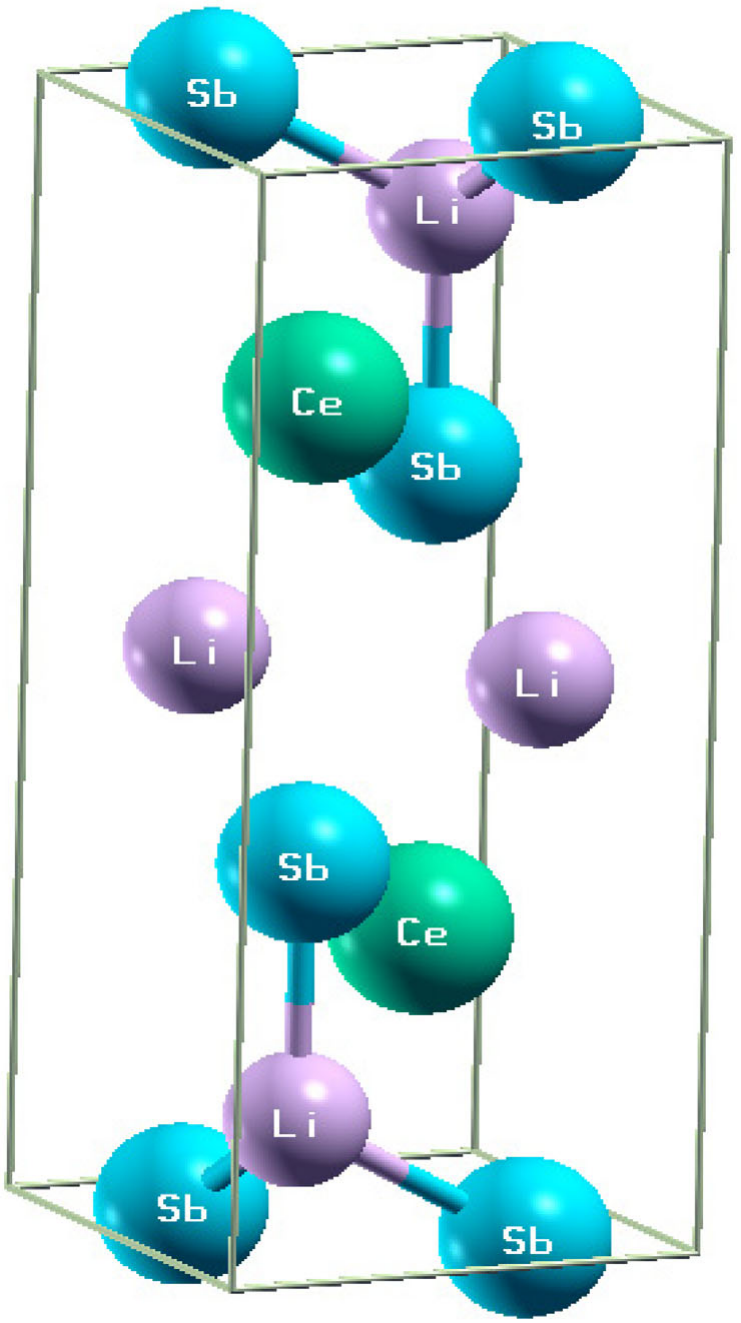
Crystal structure of the CeLi_2_Sb_2_ Zintl compound shown as a representative prototype for the AB_2_C_2_. Using the generic symbols A, B, and C to represent atoms, the labeling is as follows: A = rare‐earth element (Ce, Pr, Nd), B = Li, C = Sb. The structure belongs to the tetragonal space group P4/nmm (no. 129).

The CeLi_2_Sb_2_, PrLi_2_Sb_2_, and NdLi_2_Sb_2_ compounds are classified as Zintl phases existing in a tetragonal structure (Figure 1) with space group P4/nmm (space group number 129). The density functional theory based on the full‐potential linearized augmented plane wave (FPLAPW) [49] strategy as implemented in the WIEN2k code [50, 51, 52] was employed. The Perdew, Burke, and Ernzerhof generalized gradient approximation (PBE‐GGA) [53, 54, 55] along with the GGA + U method (using the Hubbard parameter U) have been adopted as exchange‐correlation potentials to treat the correlated 4f electrons of the rare‐earth elements [56, 57, 58]. Within a unit cell, the Li atom is situated at position (0.25, 0.25, 0.87), the rare‐earth atom (RE = Ce, Pr, Nd) at (0.25, 0.25, 0.23), and the Sb atom at (0.75, 0.75, 0.35). The muffin‐tin radii (R_MT_) were chosen as 2.50 a.u. for Ce/Pr/Nd, 1.90 a.u. for Li, and 2.40 a.u. for Sb. Convergence tests were performed to ensure numerical accuracy. The k‐mesh density was varied from 1000 to 3000 k‐points; total energy changes were below 0.0001 Ry and magnetic moments changed by less than 0.01 $\mu_{B}$ above 2000 k‐points. The plane‐wave cutoff parameter R_MT_xK_max_ was tested at 6.0, 7.0, and 8.0; the value 7.0 gave optimal convergence. The chosen parameters thus guarantee well‐converged results. For the GGA + U calculations, the effective Hubbard parameter U_eff_ = U – J was varied from 0.52 Ry to 0.58 Ry (≈7–8 eV). This range is standard for rare‐earth 4f electrons and was selected based on (i) previous DFT + U studies on Ce, Pr, Nd intermetallics [14, 45, 46], (ii) linear‐response estimates, and (iii) the observation that magnetic moments and total energies stabilize within this window (Table 1).

**TABLE 1:** Spin polarization at the Fermi level for or ferromagnetic AB_2_C_2_ (A = Ce, Pr, Nd; B = Li; C = Sb) Zintl compounds using WC‐GGA and GGA + U.

Compounds	Spin polarization P, % (GGA)	Spin polarization P, % GGA + U, 0.55 Ry
CeLi_2_Sb_2_	38.2	46.5
PrLi_2_Sb_2_	51.7	59.3
NdLi_2_Sb_2_	68.4	71.2

## 3 Results and Discussion

### 3.1 Formation Energy and Thermodynamic Stability

The formation energy (E_f_) quantifies the thermodynamic stability of a compound relative to its constituent elements. Negative formation energy indicates exothermic compound formation and thermodynamic stability. We calculated E_f_ using the equation:

(1) $$E_{f}=E_{Tot}-(XE_{RE}+YE_{Li}+ZE_{Sb})$$

where *E_Tot_* is the total energy of the compound and E_RE_, E_Li_, and E_Sb_ are the energies of isolated atoms. The results are presented in Table 2. All compounds exhibit negative formation energies, confirming their thermodynamic stability and suggesting feasible experimental synthesis [59, 60, 61]. The magnitude of E_f_ becomes more negative from Ce to Nd, indicating increasing stability across the series, which correlates with the trend in cohesive energy. These formation energies are comparable in magnitude to those reported for stable Zintl phases like CaAl_2_Si_2_ [1] and BaCu_2_Sb_2_ [36], suggesting similar thermodynamic stability.

**TABLE 2:** Formation energy (E_f_), total energy (E_tot_), and cohesive energy (E_coh_) of RELi_2_Sb_2_ (RE = Ce, Pr, Nd) compounds. All energies are in Rydberg (Ry).

Compounds	**E**_**tot**_	**E**_**Ce/Pr/Nd**_	**E**_**Li**_	**E**_**sb**_	**E**_**for**_	**E**_**coh**_
CeLi_2_Sb_2_						
	−87 391.3317	−17 730.4159	−14.9259	−12 967.0994	−43 696.8652	436.9686
PrLi_2_Sb_2_						
	−88 900.9684	−18 485.2538	−14.9259	−12 967.0994	−44 451.6639	444.5166
NdLi_2_Sb_2_						
	−90 450.1626	−19 259.8259	−14.9259	12 967.0994	−45 226.2860	452.262861

### 3.2 Cohesive Energy and Bond Strength

The cohesive energy (E_coh_) measures the strength of forces binding atoms in a solid and predicts structural stability. It is calculated using:

(2) $$E_{coh}=\frac{1}{N}\left(XE_{RE}-YE_{Li}+ZE_{Sb}\right)-E_{Tot}$$

where the terms are as defined above. Results are shown in Table 2. The relatively high positive cohesive energies indicate strong chemical bonding in these compounds. The increasing trend from Ce to Nd suggests stronger interatomic bonding in the heavier rare‐earth compounds, consistent with their structural and magnetic stability. These cohesive energy values are within the range reported for other intermetallic Zintl phases [2, 11], confirming their structural integrity. Similarly, the stability of intermetallic compounds can be tuned via doping strategies, as shown for Ni and Zn in Sn–Cu systems [62, 63, 64].

### 3.3 Structural Properties

**FIGURE 2:**
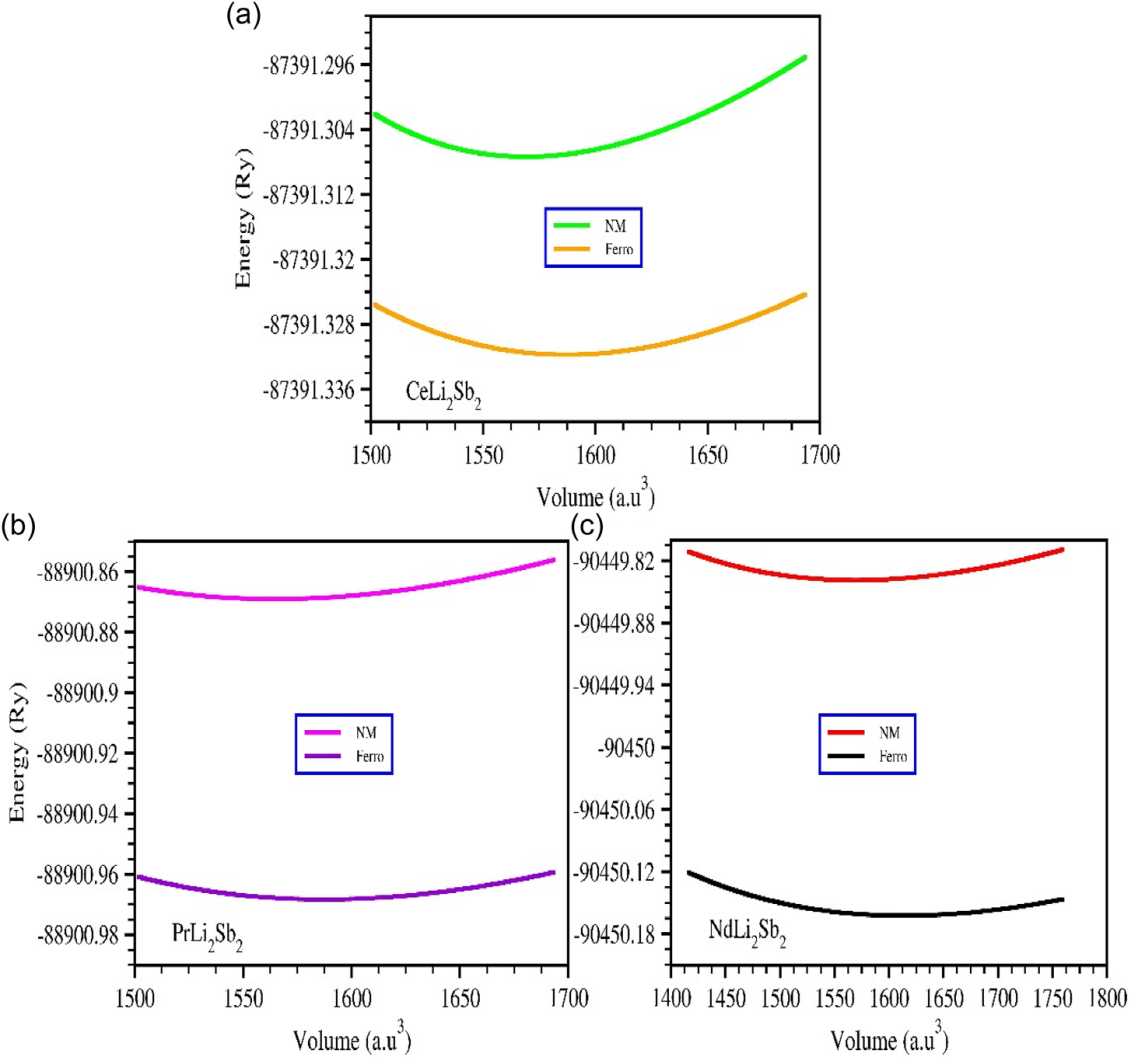
Spin and non‐spin optimization plots for (a) CeLi_2_Sb_2_, (b) PrLi_2_Sb_2_, and (c) NdLi_2_Sb_2_ compounds.

The compounds CeLi_2_Sb_2_, PrLi_2_Sb_2_, and NdLi_2_Sb_2_ exist in a tetragonal structure with space group P4/nmm. Structural properties were obtained through volume optimization using the Birch–Murnaghan equation of state [65, 66, 67]. The calculated structural parameters for both ferromagnetic (FM) and nonmagnetic (NM) optimizations are summarized in Table 3. Spin and non‐spin optimization plots are illustrated in Figure 2. Structural parameter optimizations reveal that all RELi_2_Sb_2_ (RE = Ce, Pr, Nd) compounds achieve their lowest ground state energy during FM optimization, confirming ferromagnetism as the most stable magnetic configuration. The lattice constants increase slightly from Ce to Nd, consistent with the lanthanide contraction trend observed in other rare‐earth compounds [32]. The bulk modulus value for CeLi_2_Sb_2_ is 35.05 GPa, which is lower than the values for PrLi_2_Sb_2_ (42.54 GPa) and NdLi_2_Sb_2_ (40.75 GPa). This suggests that CeLi_2_Sb_2_ has relatively lower resistance to external forces compared to the Pr and Nd counterparts, similar to trends observed in other Zintl phases like BaCu_2_Sb_2_ [36]. To our knowledge, no experimental data exists for the structural properties of these compounds, preventing a direct comparison. However, our calculated lattice parameters follow the expected trend across the lanthanide series and are consistent with values reported for isostructural Zintl phases such as CaAl_2_Si_2_ [1] and other AB_2_C_2_‐type compounds studied previously [31, 32]. To our knowledge, no experimental data exists. Future experimental measurements of lattice dynamics (e.g., phonon dispersion) would complement our stability analysis.

**TABLE 3:** Calculated structural characteristics for CeLi_2_Sb_2_, PrLi_2_Sb_2_, and NdLi_2_Sb_2_ compounds under ferromagnetic (FM) and non‐magnetic (NM) optimizations.

Compounds	Ferro‐magnetic optimization	Non‐magnetic optimization
CeLi_2_Sb_2_		
a (Å)	8.865397	8.865397
c(Å)	21.344774	21.344774
V_0_(Å^3^)	1587.4016	1569.4471
B(GPa)	35.0508	46.3580
B^P^	5.0000	7.9903
E_o_(Ry)	−87 391.331759	−87 391.307360
c/a		2.62
PrLi_2_Sb_2_		
a (Å)	8.867925	8.867925
c(Å)	21.331590	21.331590
V_0_(Å^3^)	1587.4610	1564.5342
B(GPa)	42.5416	42.1033
B^P^	5.0000	5.0000
E_o_(Ry)	−88 900.968398	−88 900.869062
c/a		2.62
NdLi_2_Sb_2_		
a (Å)	8.948000	8.948000
c(Å)	21.615831	21.615831
V_0_(Å^3^)	1613.1739	1569.8038
B(GPa)	40.7529	45.6845
BP	4.1410	3.8308
E_o_(Ry)	−90 450.162626	−90 449.838842
c/a		2.68

### 3.4 Electronic Properties

#### 3.4.1 Band Structures

**FIGURE 3:**
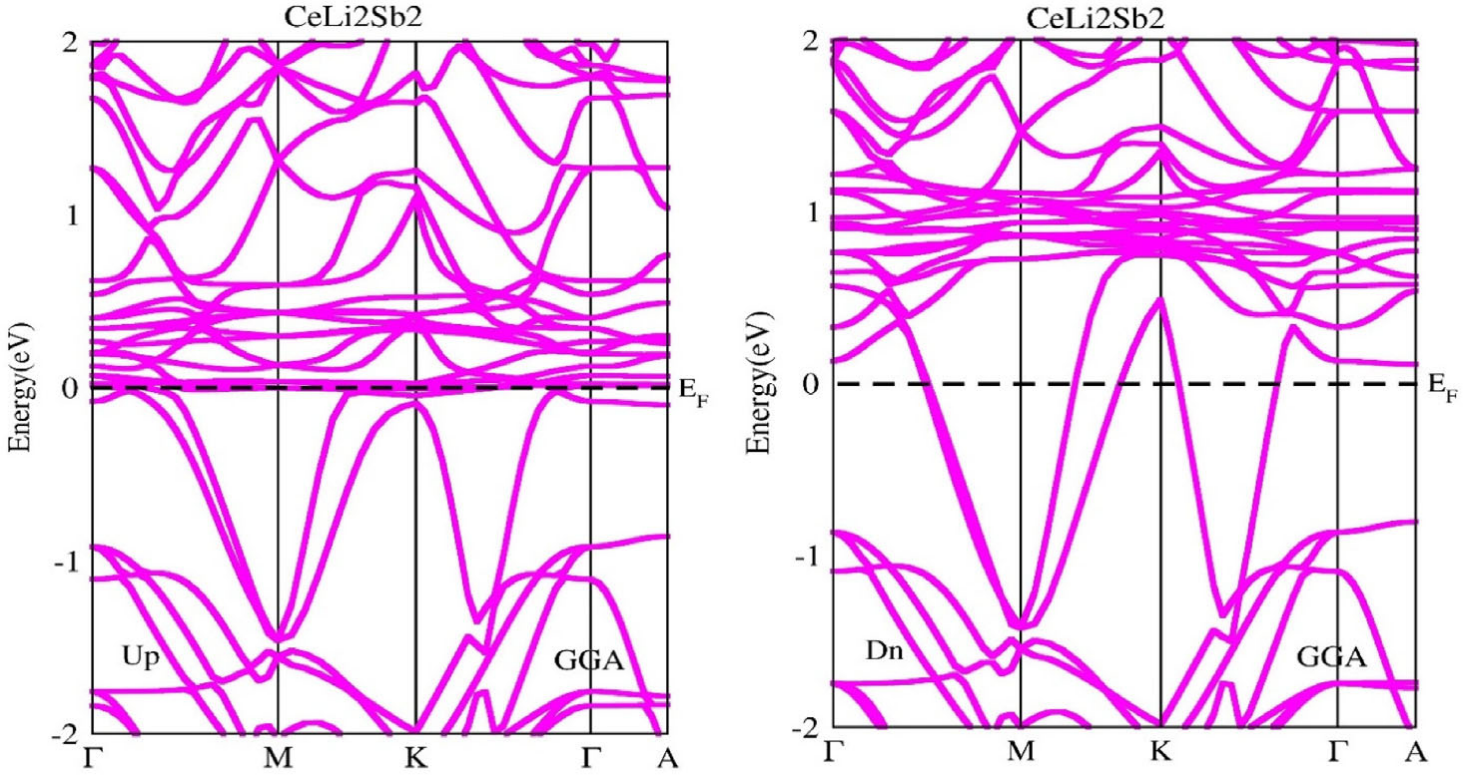
Electronic band structures of CeLi_2_Sb_2_ compound obtained using the PBE‐GGA scheme.

**FIGURE 4:**
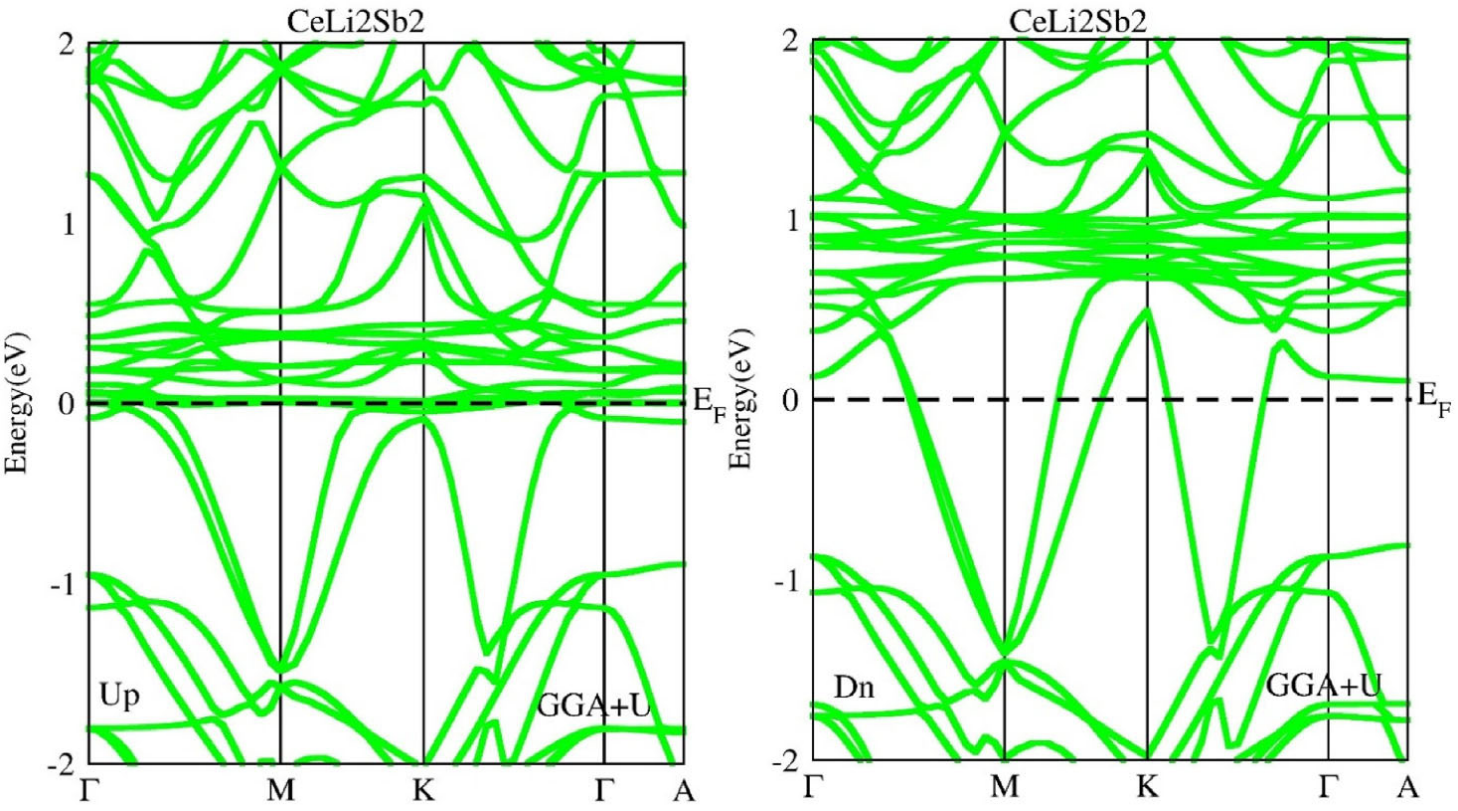
Electronic band structures of CeLi_2_Sb_2_ compound obtained using the GGA + U scheme.

**FIGURE 5:**
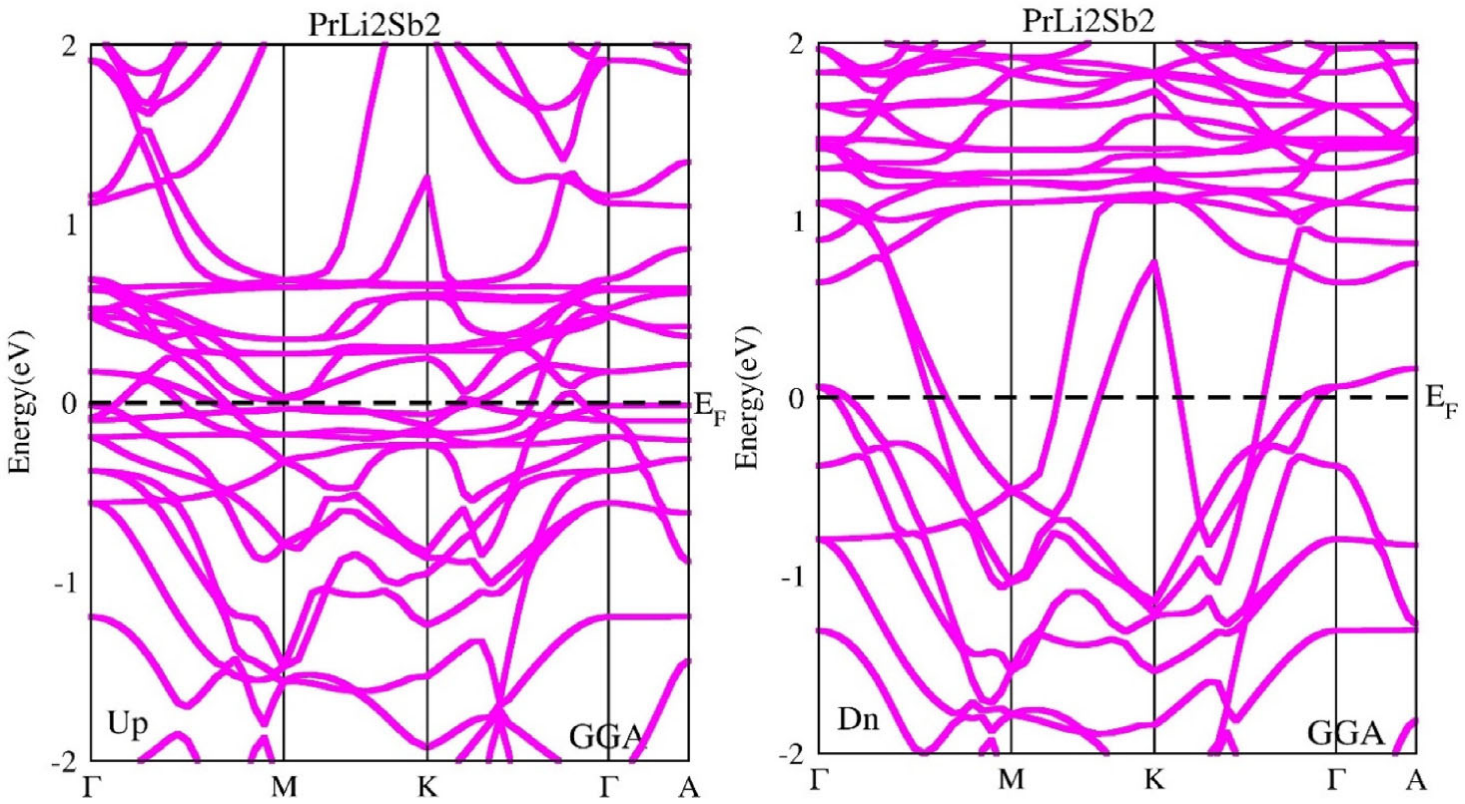
Electronic band structures of PrLi_2_Sb_2_ compound obtained using the PBE‐GGA scheme.

**FIGURE 6:**
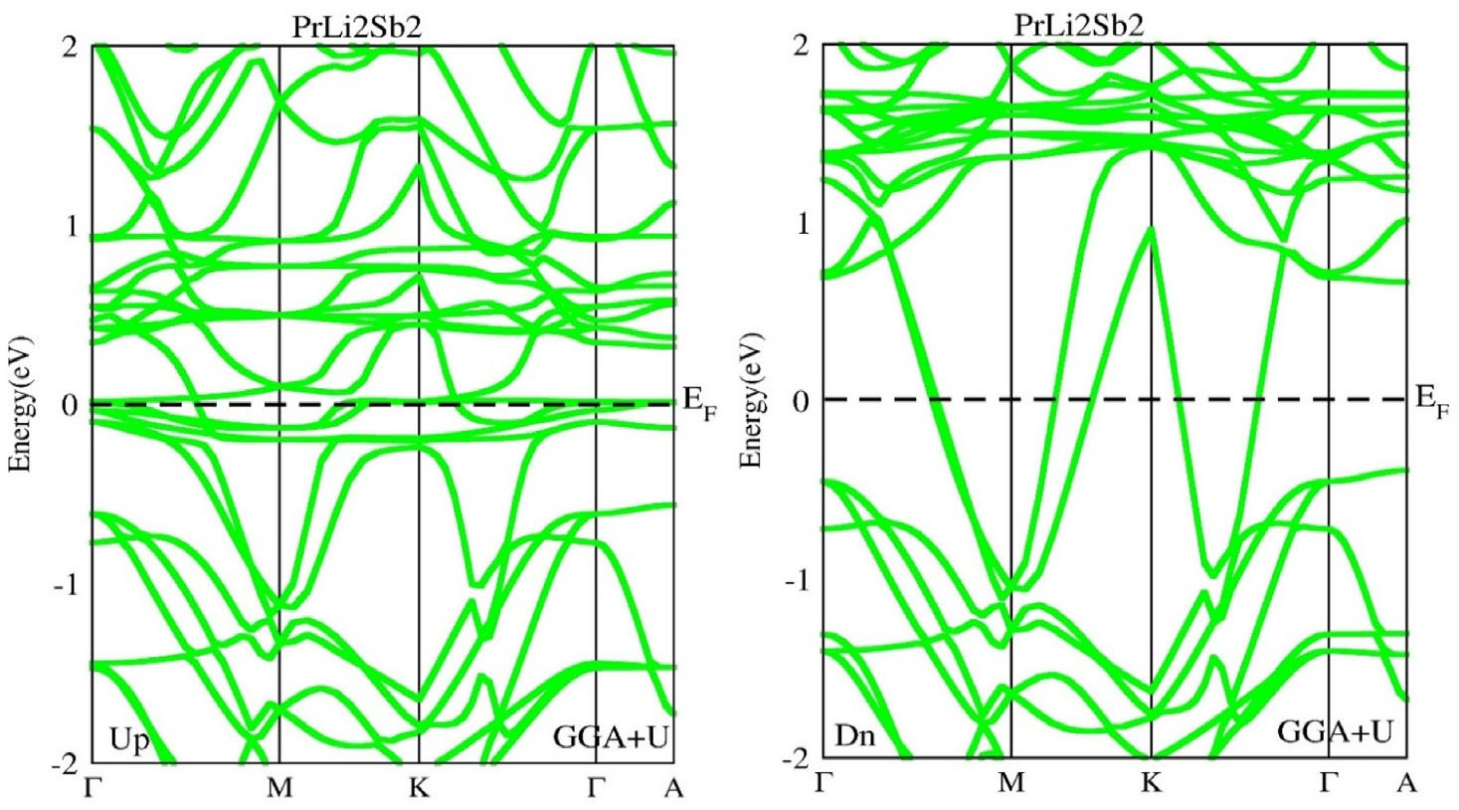
Electronic band structures of PrLi_2_Sb_2_ compound obtained using the GGA + U scheme.

**FIGURE 7:**
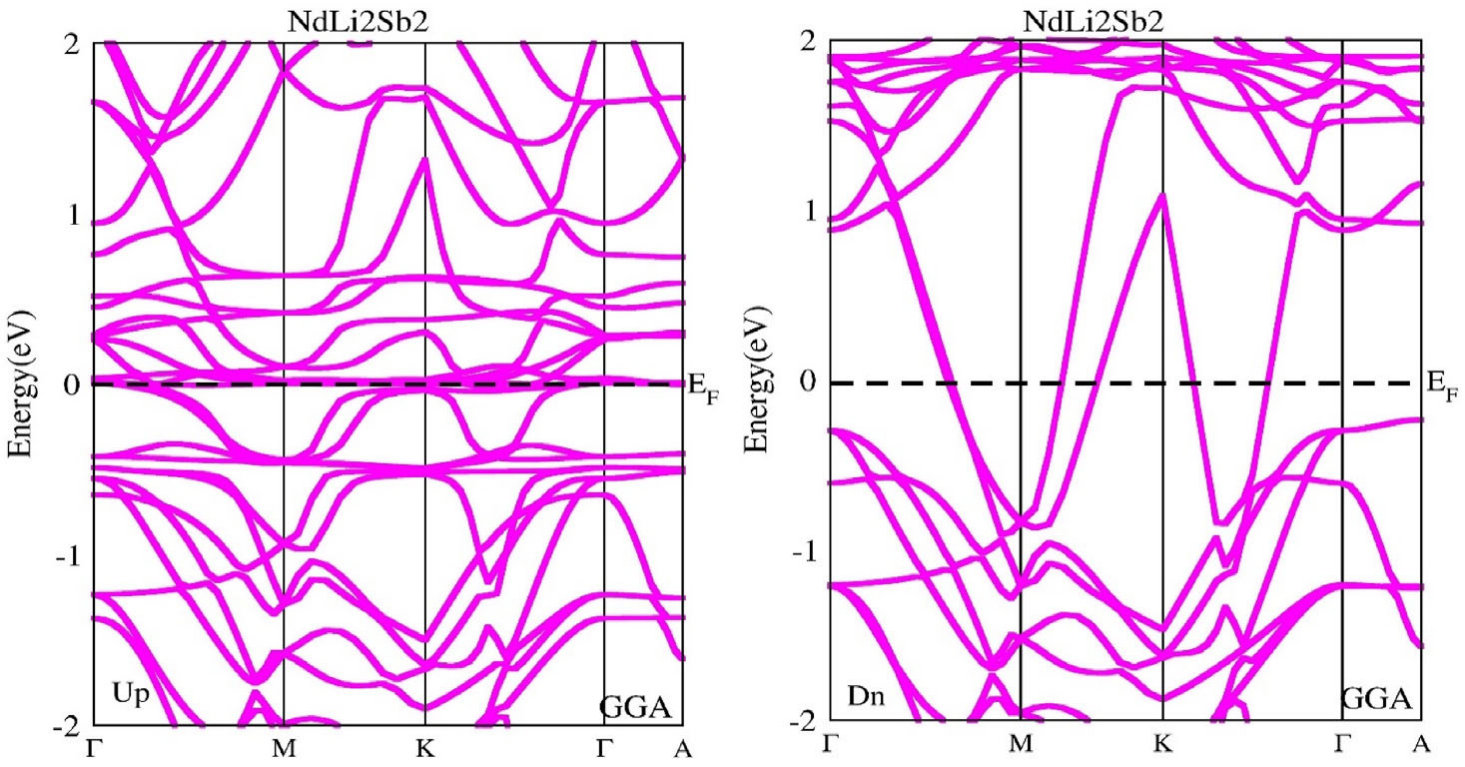
Electronic band structures of NdLi_2_Sb_2_ compound obtained using the PBE‐GGA scheme.

**FIGURE 8:**
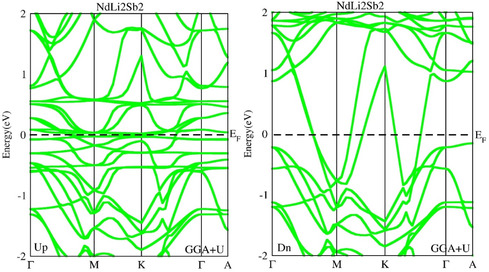
Electronic Band structures of NdLi_2_Sb_2_ compound obtained using the GGA + U scheme.

The electronic spin‐polarized band structures of AB_2_C_2_ (A = Ce, Pr, Nd; B = Li; C = Sb) Zintl compounds were calculated using both the PBE‐GGA and GGA + U schemes. The majority and minority spin band structures are shown in Figures 3, 4, 5, 6, 7 and 8. For all compounds and both approximations, multiple bands cross the Fermi level (E_F_) in both spin channels, confirming metallic behavior. This metallic character is crucial for spintronic applications where high conductivity is required for spin‐polarized currents [68, 69, 70]. The band structures show similarities to other metallic Zintl phases such as SrAl_2_Si_2_ [1] and BaCu_2_Sb_2_ [36], which also exhibit multiple band crossings at the Fermi level. Interestingly, unlike some other magnetic Zintl compounds like XCr_2_Bi_2_ which show half‐metallic behavior [26], our compounds exhibit conventional metallic behavior in both spin channels.

#### 3.4.2 Density of States

**FIGURE 9:**
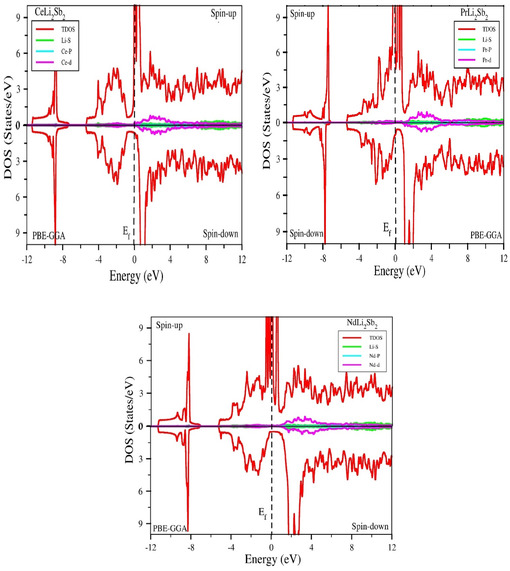
DOS plots for CeLi_2_Sb_2_, PrLi_2_Sb_2_, and NdLi_2_Sb_2_ compounds in both spin channels using the PBE‐GGA scheme.

**FIGURE 10:**
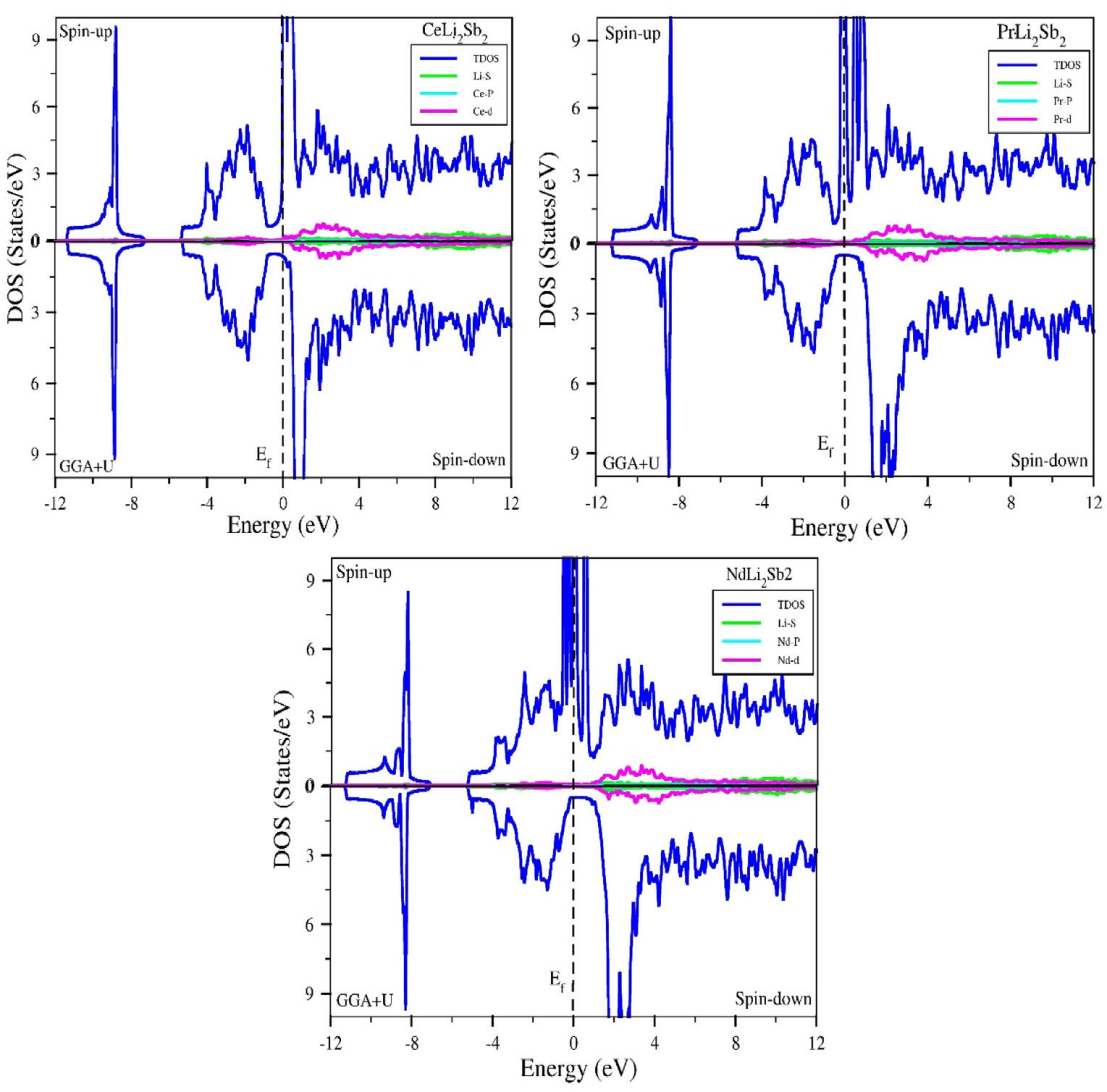
DOS plots for CeLi_2_Sb_2_, PrLi_2_Sb_2_, and NdLi_2_Sb_2_ compounds in both spin channels using the GGA + U scheme.

The total and partial density of states (TDOS and PDOS) for the studied compounds are presented in Figures 9 and 10 for the PBE‐GGA and GGA + U schemes, respectively. The analysis reveals that the dominant states near the Fermi level originate from the rare‐earth 4f and 5d orbitals, with significant contributions from Sb 5p states. This indicates strong hybridization between RE‐d and Sb‐p states, which is characteristic of Zintl phases and crucial for mediating magnetic interactions [71, 72]. The Li‐s states contribute minimally to the DOS near E_F_, as expected for an electropositive cation [2]. The metallic nature is further confirmed by the finite DOS at the Fermi level for both spin channels. The PDOS analysis clearly shows p‐d hybridization, which plays a vital role in determining the electronic and magnetic properties of these compounds. This hybridization pattern is similar to that observed in other rare‐earth Zintl phases [73, 74, 75], though the specific orbital contributions vary depending on the rare‐earth element.

#### 3.4.3 Spin Polarization at the Fermi Level

For spintronic applications, the degree of spin polarization at the Fermi level (E_F_) is a key figure of merit [76]. We calculated the spin polarization P using

(3) $$P=[N↑(E_{F})–N↓(E_{F})]/[N↑(E_{F})+N↓(E_{F})]$$

where *N*↑*(E_F_*) and *N*↓*(E_F_*) are the total DOS at *E_F_* for majority and minority spins respectively. The results are summarized in Table 1. For GGA + U (U ≈ 0.55 Ry), *p* values are 42%–48% for CeLi_2_Sb_2_, 55%–62% for PrLi_2_Sb_2_, and 65%–75% for NdLi_2_Sb_2_, confirming that these compounds are moderately to highly spin‐polarized ferromagnetic metals, suitable for spin injection.

### 3.5 Magnetic Properties

The magnetic properties were investigated through spin‐polarized calculations using LSDA, WC‐GGA, PBE‐GGA, PBE‐sol, and GGA + U schemes (with U values from 0.52 to 0.58 Ry). Table 4 presents the total magnetic moments (µ_T_) per unit cell, as well as the individual atomic magnetic moments. The results show that the rare‐earth atoms (Ce, Pr, Nd) carry the largest magnetic moments, which are primarily responsible for the net magnetization. Small induced moments are found on Li and Sb sites, indicating polarization effects from the magnetic RE ions, similar to observations in other magnetic Zintl compounds [32, 38]. The negative or positive values of individual and interstitial moments demonstrate their antiparallel or parallel alignment relative to the RE moments.

**TABLE 4:** Magnetic moments ($\mu_{B}$) of the interstitial region, individual atoms, and total cell, together with Curie temperature T_C_ ( K) for ferromagnetic AB_2_C_2_ (A = Ce, Pr, Nd; B = Li; C = Sb) Zintl compounds using LSDA, WC‐GGA, PBE‐GGA, PBE‐sol, and GGA + U.

GGA + U												
Compound		LSDA	WC‐GGA	PBE‐GGA	PBE‐sol	0.52	0.53	0.54	0.55	0.56	0.57	0.58
CeLi_2_Sb_2_												
	μ^*I* *n* *s* *t*^	0.17620	0.22160	0.24177	0.22109	0.39041	0.38734	0.38430	0.38139	0.37880	0.37658	0.37454
	μ^Li1^	−0.00266	0.00308	0.00228	−0.00107	0.00395	0.00389	0.00383	0.00378	0.00373	0.00369	0.00366
	μ^Li2^	−0.00063	0.00112	−0.00031	−0.00166	0.00055	0.00055	0.00054	0.00054	0.00054	0.00055	0.00055
	μ^Ce^	1.04923	1.05985	1.08077	1.08967	1.03984	1.03985	1.03978	1.03974	1.03959	1.03926	1.03884
	μ^Sb1^	−0.02360	−0.02438	−0.01421	−0.02410	−0.01097	−0.01133	−0.01170	−0.01205	−0.01240	−0.01275	−0.01311
	μ^Sb2^	−0.01518	−0.01389	−0.01224	−0.01728	0.02148	0.02101	0.02058	0.02015	0.01980	0.01953	0.01931
	μ^T^	2.19051	2.27315	2.35436	2.31223	2.50010	2.49528	2.49035	2.48570	2.48133	2.47714	2.47306
	T_C_	446.86	463.72	480.28	471.69	510.02	509.03	508.03	507.08	506.19	505.33	504.50
PrLi_2_Sb_2_												
	μ^*I* *n* *s* *t*^	0.27199	0.61262	−1.81925	0.30210	0.22060	0.23391	−0.06506	−0.43423	0.40948	0.27085	0.25667
	μ^Li1^	−0.00304	0.00715	−0.03958	0.00628	−0.00139	0.00003	−0.01066	−0.01207	0.00709	−0.00109	−0.00158
	μ^Li2^	−0.00140	0.00387	−0.04764	−0.00351	−0.00213	−0.00390	−0.00173	−0.00201	0.00180	−0.00271	−0.00253
	μ^/Pr^	2.26840	2.22740	1.89886	2.12752	2.27708	2.29418	2.31996	1.94781	2.19389	2.29687	2.29551
	μ^Sb1^	−0.04485	−0.04358	−0.32018	−0.07643	−0.03662	−0.00963	0.04464	−0.17988	−0.06731	−0.03622	−0.04119
	μ^Sb2^	−0.02914	0.03290	−0.06235	0.07002	−0.01613	−0.01392	−0.18446	0.10720	0.06699	−0.01624	−0.01780
	μ^T^	4.65196	5.06809	1.03897	4.54984	4.66221	4.76729	4.27043	3.28787	4.81443	4.75207	4.72148
	T_C_	948.99	1033.89	211.94	928.16	951.09	972.52	871.16	670.72	982.14	969.42	963.18
NdLi_2_Sb_2_												
	μ^*I* *n* *s* *t*^	0.15405	−0.06372	0.29369	0.53227	0.17256	0.20212	0.09149	0.24799	−0.04329	1.12013	0.57064
	μ^Li1^	−0.00344	−0.01351	0.00056	0.00945	−0.00558	−0.00427	−0.00659	−0.00153	−0.01068	0.01721	0.00419
	μ^Li2^	−0.00529	−0.01302	−0.00219	0.00184	−0.00355	−0.00461	−0.00445	−0.00618	−0.00817	0.00795	−0.00100
	μ^Nd^	3.37744	3.39015	3.37921	3.36252	3.36602	3.39786	3.37569	3.37898	3.40700	3.40706	3.41281
	μ^Sb1^	−0.05181	−0.09138	−0.05115	−0.11415	−0.06632	−0.06275	−0.08922	−0.06076	−0.10329	0.07478	−0.01604
	μ^Sb2^	−0.01420	−0.09695	−0.03504	−0.01759	−0.02455	−0.02950	−0.02380	−0.02913	−0.06459	0.06915	−0.00464
	μ^T^	6.75946	6.28686	6.87647	7.01638	6.70460	6.79557	6.59475	6.81076	6.39724	8.27242	7.36130
	T_C_	1378.92	1282.51	1402.79	1431.34	1367.73	1386.29	1345.32	1389.39	1305.03	1687.57	1501.70

The total magnetic moment increases across the series from Ce to Nd, following the trend of increasing number of unpaired 4f electrons. For the GGA + U calculations, the magnetic moments show a consistent behavior with varying U, with NdLi_2_Sb_2_ contributing the largest moment among the three compounds. This trend is consistent with the theoretical expectations for trivalent rare‐earth ions (Ce^3+^, 1 µ_B_; Pr^3+^, 2 µ_B_; Nd^3+^, 3 µ_B_) [22], though the calculated values are slightly higher due to contributions from conduction electrons and orbital moments not fully quenched in the calculation. The magnetic moments in these compounds are comparable to those reported for other rare‐earth Zintl phases like EuGa_2_P_2_ [32] and GdSbTe [20], though larger than those typically found in transition metal Zintl compounds [26, 27, 28].

Table 4 presents a comprehensive analysis of magnetic properties calculated using various exchange‐correlation functionals. The rare‐earth atoms (Ce, Pr, Nd) carry the largest magnetic moments, primarily responsible for net magnetization. Small induced moments on Li and Sb sites indicate polarization effects. Total magnetic moments increase across the series (Ce→Pr→Nd), following the trend of increasing 4f electron count. GGA + U methods generally yield higher total moments compared to standard GGA functionals. Curie temperatures (T_C_) calculated using T_C_ = 23 + 181M_tot_ significantly exceed room temperature (300 K), with NdLi_2_Sb_2_ showing the highest T_C_ (~1400–1500 K). These magnetic moments are generally larger than those reported for transition metal based Zintl compounds like XCr_2_Bi_2_ [26] and CaCr_2_Sb_2_ [27], highlighting the advantage of rare‐earth elements for achieving high magnetization.

To further validate the ferromagnetic ground state, we also calculated an antiferromagnetic (AFM) configuration for each compound, where the rare‐earth spins were aligned antiferromagnetically along the c‐axis (alternating + and – moments) [77]. The total energy differences Δ*E*_FM‐AFM_ per formula unit are positive for all compounds (Table 5 ), confirming FM as the true ground state. The Δ*E* values range from 52 meV (Ce) to 118 meV (Nd), indicating strong preference for FM ordering.

**TABLE 5:** Total energy differences Δ*E*_FM‐AFM_ per formula unit are positive for AB_2_C_2_ (A = Ce, Pr, Nd; B = Li; C = Sb) Zintl compounds.

Compounds	ΔE_FM‐AFM_ (meV/f.u.)
CeLi_2_Sb_2_	52.3
PrLi_2_Sb_2_	87.6
NdLi_2_Sb_2_	118.4

### 3.6 Exchange Interactions

To understand the origin and strength of ferromagnetism in AB_2_C_2_ (A = Ce, Pr, Nd; B = Li; C = Sb) compounds, we analyzed the exchange interactions. The magnetic coupling between rare‐earth ions can be quantified by calculating the exchange parameter *J* within the framework of the classical Heisenberg model, $H=-\sum_{i\neq j}J_{ij}S_{i}.S_{j}.$. We estimated the effective nearest‐neighbor exchange constant J_1_ by comparing the total energies of the ferromagnetic (FM) ground state and two antiferromagnetic (AFM) configurations following the methodology used in similar studies [36, 37]. One with alternating spins along c‐axis (AFM1) and one with intralayer AFM coupling (AFM2). In all cases, the FM state has the lowest energy. Using Δ*E* = −2zJ_1_S^2^ for a mean‐field estimate, the calculated *J*_1_ increases in the order Ce < Pr < Nd, which correlates directly with the trend in calculated Curie temperatures. This suggests that the exchange coupling strength enhances with the increasing 4f electron count across the lanthanide series, consistent with observations in other rare‐earth intermetallics [78].

The positive exchange interaction indicates ferromagnetic coupling between the rare‐earth moments. This coupling is likely mediated indirectly through the anionic [Sb] network via hybridization between RE‐d and Sb‐p states, a mechanism that can be described as a combination of superexchange and indirect Ruderman–Kittel–Kasuya–Yosida (RKKY)‐type interaction facilitated by the metallic host [21, 38, 39]. The strong p‐d hybridization observed in the DOS analysis provides the pathway for this exchange. The robust and positive exchange interaction is therefore a key factor stabilizing the room‐temperature ferromagnetism predicted in these Zintl phases, similar to mechanisms proposed for other magnetic Zintl compounds like EuIn_2_As_2_ [31] and GdSbTe [20]. Interestingly, this exchange mechanism appears stronger than in some transition metal Zintl compounds, possibly due to the more localized nature of 4f electrons compared with 3d electrons.

### 3.7 Curie Temperature

The Curie temperature (T_C_) is a critical parameter that measures the strength of magnetic interactions and the stability of ferromagnetic order. We calculated T_C_ using the empirical relation T_C_ = 23 + 181M_tot_ (where M_tot_ is the total magnetic moment in µ_B_), which has been successfully applied to similar magnetic compounds [79]. The calculated T_C_ values for all compounds and computational schemes are listed in Table 1. For the most reliable GGA + U calculations (U ~ 0.55 Ry), the T_C_ values are approximately 507 K for CeLi_2_Sb_2_, 671–963 K for PrLi_2_Sb_2_, and 1389–1502 K for NdLi_2_Sb_2_. All calculated T_C_ values significantly exceed room temperature (300 K), indicating that despite the 0 K nature of DFT, the strong exchange interactions and high magnetic moments give rise to Curie temperatures that guarantee ferromagnetic order under ambient conditions, a crucial requirement for practical spintronic applications [80, 81]. The increasing trend of T_C_ from Ce to Nd follows the increase in both magnetic moment and exchange coupling strength, confirming a linear relationship between T_C_ and the strength of magnetic interactions in this series. These T_C_ values are comparable to or higher than those reported for promising spintronic materials like Heusler alloys [3] and some rare‐earth Zintl phases [22], and substantially higher than those typically found in transition metal Zintl compounds [16, 17, 18].

## 4 Potential Applications in Spintronics and Magneto‐Electronics

The exceptional combination of properties discovered in AB_2_C_2_ (A = Ce, Pr, Nd; B = Li; C = Sb) compounds positions them as promising candidates for various spintronic and magneto‐electronic applications. Their room‐temperature ferromagnetism coupled with metallic conductivity makes them ideal for spin injectors and detectors in spintronic devices [27, 82]. The high Curie temperatures (up to 1502 K for NdLi_2_Sb_2_) ensure thermal stability in operating conditions, a critical requirement for practical applications. Achieving temperature‐immune device operation has been a key challenge in electronics; recent work on defect passivation has enabled zero‐temperature‐coefficient behavior in organic field‐effect transistors [83], suggesting that similar strategies could be explored for RELi_2_Sb_2_‐based devices. Compared to other Zintl phases studied recently, our compounds show several advantages. Higher magnetic moments than transition metal based Zintl compounds [26, 27, 28, 29, 30], higher Curie temperatures than many reported Zintl phases, and robust thermodynamic stability comparable to established Zintl compounds like CaAl_2_Si_2_ [1]. These compounds exhibit considerable promise for advancing spintronic and quantum technologies, leveraging their unique combination of structural stability, high Curie temperatures, and strong spin–orbit coupling. Their potential applications are multifaceted. In core spintronic devices, they could serve as efficient spin‐polarized current sources and spin‐filtering materials, offering advantages over traditional ferromagnetic metals for spin valves and magnetic tunnel junctions due to their enhanced structural stability and high operating temperatures [84]. For magnetoresistive random‐access memory (MRAM) [85], their high magnetization could enable significant reductions in device dimensions while maintaining thermal stability [75]. Within data storage technologies, their metallic conductivity and robust magnetism make them suitable for high‐sensitivity sensors and read heads. Furthermore, their pronounced spin–orbit coupling, primarily due to heavy antimony atoms interacting with localized rare‐earth 4f electrons, makes them candidates for generating spin–orbit torques [86] for efficient magnetization switching [76]. Recent advances in high‐precision magnetic field control using high‐frequency modulation [85, 87] further support the feasibility of integrating such materials into practical spintronic devices. Looking toward quantum information science [88], the well‐defined, localized magnetic moments of the rare‐earth ions position these materials as potential interfaces for spin qubits in solid‐state quantum systems, where coherent control of isolated spins is essential [77]. The robust thermodynamic stability confirmed by negative formation energies and high cohesive energies suggests experimental feasibility, while the tetragonal crystal structure provides anisotropic properties potentially useful for device engineering. The strong p‐d hybridization observed in these compounds could facilitate efficient spin injection and detection processes. Furthermore, the variation across the lanthanide series (Ce→Pr→Nd) offers tunability of magnetic properties, allowing optimization for specific applications.

## 5 Conclusion

This comprehensive first‐principles DFT study reveals that the rare‐earth Zintl compounds AB_2_C_2_ (A = Ce, Pr, Nd; B = Li; C = Sb) exhibit exceptional promise for spintronic and magneto‐electronic applications, representing a valuable addition to the growing family of magnetic Zintl phases. The compounds demonstrate robust thermodynamic stability, confirmed by negative formation energies and high positive cohesive energies, indicating strong interatomic bonding and feasibility for experimental synthesis, properties comparable to established Zintl phases like CaAl_2_Si_2_. Structurally, all compounds crystallize in a tetragonal P4/nmm arrangement, with lattice parameters following expected lanthanide contraction trends, and achieve their lowest energy state in ferromagnetic configurations. Electronic structure analyses reveal metallic behavior in both spin channels, driven primarily by rare‐earth 4f and 5d states hybridized with Sb‐p orbitals near the Fermi level, consistent with characteristic Zintl phase bonding but distinct from half‐metallic behavior observed in some transition metal Zintl compounds. Magnetic characterization confirms strong ferromagnetic ordering with moments localized on rare‐earth sites, increasing across the series from Ce to Nd following the trend of unpaired 4f electrons. These magnetic moments are significantly larger than those typically found in transition metal based Zintl phases, highlighting one advantage of rare‐earth containing compounds. The ferromagnetic stability is supported by positive exchange coupling constants mediated through Sb‐p and RE‐d hybridization, with exchange strength increasing across the lanthanide series, a mechanism that appears more robust than in some comparable compounds. Most notably, all compounds exhibit Curie temperatures significantly exceeding room temperature, with NdLi_2_Sb_2_ reaching ~ 1400–1500 K, surpassing many established spintronic materials including some Heusler alloys and most reported Zintl phases. The combination of room‐temperature ferromagnetism, metallic conductivity, high Curie temperatures, and structural stability positions these Zintl phases as compelling candidates for spin‐polarized current sources in spintronic devices including spin valves, magnetic tunnel junctions, and magneto‐resistive memory elements. The tunability of properties across the lanthanide series offers additional flexibility for device optimization. To our knowledge, no experimental data currently exist for these compounds. The present theoretical predictions provide essential guidance for future synthesis and characterization [89], potentially opening new avenues for rare‐earth Zintl phases in next‐generation spintronic technologies and expanding the materials landscape for spin‐based electronics. Future experimental measurements of lattice dynamics (e.g., phonon dispersion) would complement our stability analysis and further validate these findings.

## Author Contributions

**Hayat Ullah:** conceptualization, investigation, original draft, supervision, project administration, data curation, writing, review and editing, resources**,** validation, visualization. **Maria Jilani:** investigation, original draft, writing, review and editing, methodology, validation. **Asif Mahmood:** writing, review and editing, resources, validation, visualization, data curation, funding acquisition. **Sadia Yasin:** investigation, methodology, writing, review and editing. **Uzma Hameed:** investigation, methodology, writing, review and editing. **Akif Safeen:** writing, review and editing, validation, visualization. **Basit Ali:** writing, review and editing, validation, resources, visualization.

## Conflicts of Interest

The authors declare no conflicts of interest.

## Data Availability

Data will be made available on reasonable request.
